# The Left Main Complication of the Bentall’s Procedure

**DOI:** 10.4021/cr285w

**Published:** 2014-01-02

**Authors:** Malcolm Anastasius, Graham Hillis, John Yiannikas

**Affiliations:** a Cardiology Department, Concord Repatriation General Hospital, Australia; b The George Institute for Global Health, University of Sydney, Australia

**Keywords:** Aorta/aortic, Aortic operation, Aortic root, Aortic valve, Replacement

## Abstract

We present two interesting cases of critical left main stenosis following the Bentall’s procedure, with each case having a different outcome. There will also be brief discussion of the treatment for this complication.

## Introduction

The Bentall’s procedure involves a composite graft and coronary artery reimplantation to treat both an aneurysm of the aorta and aortic valve disease. We have previously reported ostial coronary pseudoaneurysm formation in a consecutive series of patients following Bentall’s surgery [1, 2]. This condition is considered to be mostly benign. However, critical left main stenosis following this surgical procedure is rare. Both cases of critical left main stenosis involve patients with bicuspid native aortic valves with associated aortopathy; however, one patient presented with aortic regurgitation and the other aortic stenosis.

## Case Report

### Case 1

A 54-year-old gentleman presented with general fatigue. Investigation 15 years previously revealed mild aortic regurgitation, a bicuspid aortic valve and a dilated aortic root. Physical examination now revealed a moderately collapsing pulse, an ejection click along the left sternal edge and a moderately long aortic regurgitation murmur. Echocardiography confirmed severe aortic regurgitation, with a bicuspid aortic valve, a severely dilated ascending aorta and normal left ventricular function. A CT aortogram demonstrated dilatation of the ascending aorta of up to 58 mm extending to the arch.

The patient was subsequently referred for surgery with the Bentall’s procedure, using a Freestyle aortic root replacement. The root was dissected, coronary ostia were mobilized, aortic leaflets were excised, annulus was sized and then the Freestyle valve conduit was sutured in place. The coronary ostia were reimplanted with proline suture material, with no use of tissue glue. The patient recovered well with no immediate complications. Follow-up transthoracic echocardiography showed good left ventricular function with a normally functioning prosthetic aortic valve. The aortic root graft appeared normal and the coronary anastomoses did not show significant enlargement.

**Figure 1:**
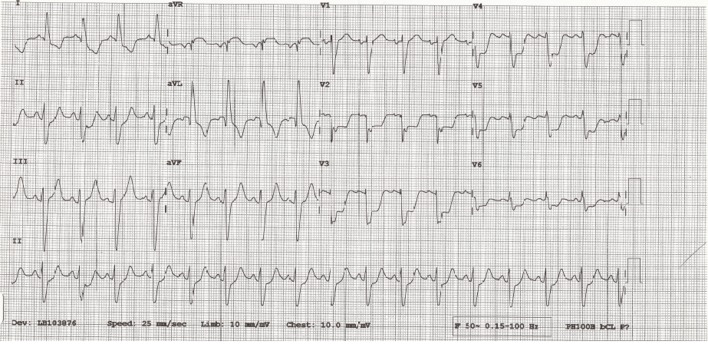
12-lead ECG showing widespread ST depression and ST elevation in aVR.

**Figure 2:**
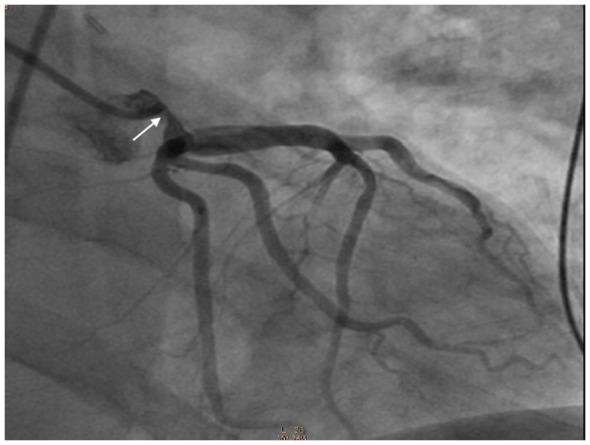
Coronary angiogram depicting the left main stenosis.

Eight weeks following the operation, the patient presented to hospital with severe chest pain and acute pulmonary edema. The electrocardiogram demonstrated profound widespread ST segment depression and ST elevation in aVR (Fig. 1). High sensitivity troponin rose to 5,400 ng/L. An urgent coronary angiogram demonstrated critical stenosis in the proximal left main coronary artery, not involving the ostial insertion (Fig. 2). The patient underwent coronary artery bypass grafting with a left internal mammary graft to the left anterior descending artery. Following the second operation transthoracic echocardiography showed moderate to severe left ventricular systolic dysfunction with akinesis of the anterior wall, interventricular septum and left ventricular apex.

Six months following the event, he remains stable and well and has returned to part-time work.

### Case 2

A 59-year-old gentleman presented originally in 2009 for routine health assessment prior to competing in the New York marathon. Examination revealed a systolic murmur and an echocardiogram indicated moderate to severe aortic stenosis. Being asymptomatic, the patient was observed carefully with regular clinical review. In May 2010, while still asymptomatic, transthoracic echocardiography indicated a heavily calcified aortic valve with moderate to severe stenosis, mild concentric left ventricular hypertrophy with normal systolic function. In addition, the scan demonstrated marked dilatation of the ascending thoracic aorta with a leading edge to leading edge diameter of 5 cm.

Given the combination of moderate to severe aortic stenosis and a dilated ascending aorta, the patient underwent aortic root and ascending aorta replacement with a hemi arch replacement. The left and right coronary ostia were mobilized with excision of the calcified bileaflet valve. The root was replaced using a Medtronic Freestyle 27 mm graft. The left main coronary artery was then reimplanted with 5/0 Prolene suture material. Progress echocardiography demonstrated normal function of the prosthetic ascending aorta and prosthetic aortic root/valve, mild left ventricular wall hypertrophy with overall normal systolic function.

**Figure 3:**
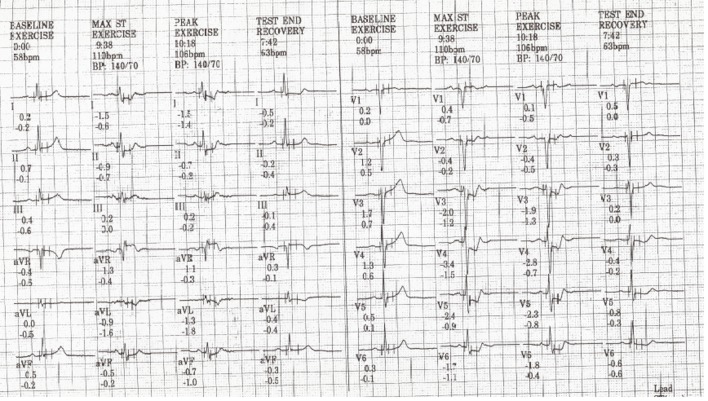
Exercise ECG showing inferolateral ST segment depression at peak exercise.

**Figure 4:**
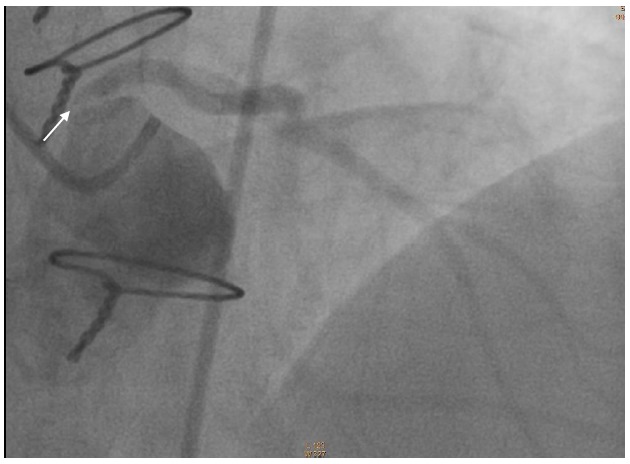
Coronary angiogram with a non-selective injection of the left coronary system, showing left main stenosis.

Twelve months following surgery, he presented with exertional dyspnea. Exercise stress testing with the Bruce protocol to a time of 8:30 min revealed 2 mm of downsloping ST segment depression maximal in V4 (Fig. 3). Subsequently, a myocardial perfusion scan demonstrated moderately extensive anterolateral ischemia. Following his strongly positive stress test he went for urgent coronary angiography. The left main vessel could not be selectively engaged but non-selective sinus injections (Fig. 4) suggested a very tight ostial stenosis that was confirmed on a CT coronary angiogram. He then underwent coronary artery bypass grafting with an LIMA to LAD graft. Almost 2 years following the second operation he remains well.

## Discussion

The Bentall’s procedure is an important surgical procedure that prolongs survival and improves symptoms with reported survival of 91.7% at 10 years [3].

Left main coronary artery stenosis resulting from the Bentall’s procedure is a life-threatening but rarely reported complication.

### Potential mechanisms

The presumed mechanisms for ostial left main stenosis following the Bentall’s procedure include: an inflammatory fibrous reaction to the suture material or tissue glue used during reimplantation of the coronary arteries, suture methods that result in kinking or stretching of the vessel or a result of instrumentation of the coronary ostia during the induction of cardioplegia [4]. It may be difficult to predict individual patient susceptibility to the inflammatory reaction. It is also possible that any fibrous inflammatory reaction may increase the risk of restenosis following PCI but the evidence for this is lacking.

### Management of left main stem stenosis following the Bentall’s procedure

The optimal management of left main stem stenosis in this setting is unclear. Coronary artery bypass grafting is recommended for the treatment of unprotected left main disease according to the American Heart Association guidelines and is the usual treatment of choice for left main stem stenosis following a Bentall’s procedure. Trivi et al [5] have reported left main stenosis 7 weeks after the Bentall’s procedure, initially treated percutaneously with a bare metal stent. Six months after PCI non-invasive testing revealed extensive myocardial ischemia and repeat angiography confirmed severe left main in-stent restenosis. The patient underwent off pump coronary artery bypass and remained symptom free at 24 months. This successful outcome was also true for our patient in case 2 managed with CABG, having presented with stable angina following the Bentall’s procedure.

Our patient in case 1 presented more acutely with left ventricular dysfunction that persisted after CABG; perhaps PCI should be more strongly considered in this acute setting. There are reports of successful short- to medium-term results from PCI. Lelasi et al [6] reported a case of a Bentall’s operation with prosthetic aortic valve replacement complicated by iatrogenic post-anastomotic left main ostial stenosis: the patient presented with exertional angina 1 month after the operation and subsequently underwent percutaneous coronary intervention with deployment of a sirolimus-eluting stent in the left main coronary artery ostium. The procedure was effective with good clinical results at 18 months.

Bernelli et al [7] also describe a case successfully treated with PCI (BMS) with no cardiac events at 11 months. Thus, PCI may be an effective means of revascularisation, especially in more acute and unstable patients [8] and particularly given the relatively high rates of perioperative infarction, morbidity and death observed following CABG in patients with a prior Bentall’s procedure [7].

In summary, the Bentall’s procedure is a well proven operation with excellent long-term results [3]. It may, however, be complicated by proximal stenosis of the reimplanted coronary arteries. This may be life-threatening and can be difficult to treat. Clinicians should be aware of this rare but important complication.
